# Resources and Costs for Microbial Sequence Analysis Evaluated Using Virtual Machines and Cloud Computing

**DOI:** 10.1371/journal.pone.0026624

**Published:** 2011-10-19

**Authors:** Samuel V. Angiuoli, James R. White, Malcolm Matalka, Owen White, W. Florian Fricke

**Affiliations:** 1 Institute for Genome Sciences (IGS), University of Maryland Baltimore, Baltimore, Maryland, United States of America; 2 Center for Bioinformatics and Computational Biology, University of Maryland, College Park, Maryland, United States of America; Baylor College of Medicine, United States of America

## Abstract

**Background:**

The widespread popularity of genomic applications is threatened by the “bioinformatics bottleneck” resulting from uncertainty about the cost and infrastructure needed to meet increasing demands for next-generation sequence analysis. Cloud computing services have been discussed as potential new bioinformatics support systems but have not been evaluated thoroughly.

**Results:**

We present benchmark costs and runtimes for common microbial genomics applications, including 16S rRNA analysis, microbial whole-genome shotgun (WGS) sequence assembly and annotation, WGS metagenomics and large-scale BLAST. Sequence dataset types and sizes were selected to correspond to outputs typically generated by small- to midsize facilities equipped with 454 and Illumina platforms, except for WGS metagenomics where sampling of Illumina data was used. Automated analysis pipelines, as implemented in the CloVR virtual machine, were used in order to guarantee transparency, reproducibility and portability across different operating systems, including the commercial Amazon Elastic Compute Cloud (EC2), which was used to attach real dollar costs to each analysis type. We found considerable differences in computational requirements, runtimes and costs associated with different microbial genomics applications. While all 16S analyses completed on a single-CPU desktop in under three hours, microbial genome and metagenome analyses utilized multi-CPU support of up to 120 CPUs on Amazon EC2, where each analysis completed in under 24 hours for less than $60. Representative datasets were used to estimate maximum data throughput on different cluster sizes and to compare costs between EC2 and comparable local grid servers.

**Conclusions:**

Although bioinformatics requirements for microbial genomics depend on dataset characteristics and the analysis protocols applied, our results suggests that smaller sequencing facilities (up to three Roche/454 or one Illumina GAIIx sequencer) invested in 16S rRNA amplicon sequencing, microbial single-genome and metagenomics WGS projects can achieve cost-efficient bioinformatics support using CloVR in combination with Amazon EC2 as an alternative to local computing centers.

## Introduction

Genome sequencing has found widespread applications, including basic science, biosafety and biomedical research, and is expected to become part of the service sector, e.g. in the form of personalized health care [1]–[3]. The popularity of genomics applications has largely been driven by the introduction of new sequencing technologies that offer increasing sequencing throughput at a decreasing cost per nucleotide. As third-generation sequencing platforms [4] are becoming available, the cost of sequence generation is likely to decrease even further. Moreover, the introduction of “benchtop” sequencing that aims at integrating medium-scale, affordable sequence generation into the standard laboratory equipment [5] is following this decentralization trend where sequencing facilities are becoming available for any size laboratory. As a result, genomics projects no longer depend on the large sequencing centers for sequence generation.

As production of sequence data continues to expand, sequence processing and bioinformatics is increasingly becoming a bottleneck for utilizing genomics approaches. So far, the decentralization of sequence production has not been accompanied by a simultaneous decentralization in computational resources and bioinformatics expertise [6]. The generation of new sequence data is increasing faster than the capacity to computationally analyze it [7], making the feasibility and affordability of future genomics projects increasingly dependent on bioinformatics components rather than on sequence generation. The resulting “bioinformatics bottleneck” describes the problem where the time and cost of basic sequence analysis may far exceed the costs of sequence generation for many researchers.

In this context, cloud computing provides an attractive model with a recognized potential for genomics and bioinformatics to meet the increasing demands for decentralized large-scale computational resources [8]. Following the definition of the National Institute of Standards and Technology (NIST), cloud computing is a “model for enabling convenient, on-demand network access to a shared pool of configurable computing resources (e.g., networks, servers, storage, applications, and services) that can be rapidly provisioned and released with minimal management effort or service provider interaction” (http://www.nist.gov/itl/cloud/upload/cloud-def-v15.pdf).

A key technology available with the cloud is the Virtual Machine (VM). A virtual machine is an operating system that can be pre-packaged with all software needed for a particular analysis. Critically, the VM is portable and can be deployed across institutions and platforms, including desktops, laptops, servers, and remote clouds. The use of VMs aims to overcome difficulties distributing analysis software that have numerous dependencies and hinders usage. This not only addresses technical challenges of deployment and installation but also enables performance comparisons and ultimately cost comparisons across institutions and architectures.

Cloud computing plus virtualization provides a model to develop and evaluate the computational infrastructure necessary for bioinformatics processing. The availability of cloud computing platforms with transparent pricing has generated an opportunity to attach real dollar costs to bioinformatics workflows and to model the associated costs for genomics applications. The Amazon Elastic Compute Cloud (EC2; http://aws.amazon.com/ec2/) provides on-demand compute (priced per CPU hour) and charges additionally for network transfers to and from the cloud (bandwidth priced per GB) and persistent data storage (priced per GB and per month). Academic clouds are also emerging that aim to offer similar services at no cost to academic researchers (e.g., DIAG, http://diag.igs.umaryland.edu/; Magellan, http://magellan.alcf.anl.gov/).

While there is considerable enthusiasm in the bioinformatics community about cloud computing [7]–[10], only a few tools and examples are available [7], [11]–[14] that demonstrate the usability of cloud services to support large-scale sequence processing. Bioinformatics case studies have been published with varying results, e.g., favoring cloud-based over local computing in both performance and cost for microarray-based transcriptomic analysis [15] or demonstrating comparable performance parameters for cloud-based and local computing and cost advantages of local executions for metagenomics BLAST analysis [14].

In order to initiate, budget and manage genomics projects the following questions need to be considered and adequately addressed beforehand: (i) What are the available methods to analyze the sequence data in order to generate publishable results in standards-conforming formats? (ii) What are the required computational requirements? (iii) What are the real dollar costs to perform the analysis? (iv) Given the amount of sequence data to be analyzed, does it make more sense to use Infrastructure as a Service (IaaS) models, such as the Amazon EC2 cloud, or to invest in a local grid network? The work presented here is intended to address these questions and provide guidelines for researchers, service providers and funding agencies, who invest in microbial genomics projects.

To evaluate the requirements for common bioinformatics applications in microbial genomics, we utilize the Cloud Virtual Resource (CloVR) package (http://clovr.org/) [16]. This software consists of a single virtual machine (CloVR VM), which contains pre-installed and pre-configured open source programs bundled into fully automated sequence analysis pipelines. CloVR supports a broad variety of small to large-scale microbial genomics applications of current and next-generation sequencing platforms with four automated pipelines: (i) 16S rRNA-based microbial community composition analysis of Sanger and 454 sequence data (CloVR-16S) [17]; (ii) taxonomic and functional community composition analysis of metagenomic whole-genome shotgun (WGS) sequence data (CloVR-Metagenomics) [18]; (iii) bacterial single-genome WGS Sanger, 454 or Illumina sequence assembly and annotation using the IGS Annotation Engine (CloVR-Microbe) [19]; and (iv) large-scale BLAST searches of Sanger, 454 or Illumina sequence data (CloVR-Search).

## Methods

### Analysis protocols

**Figure 1:**
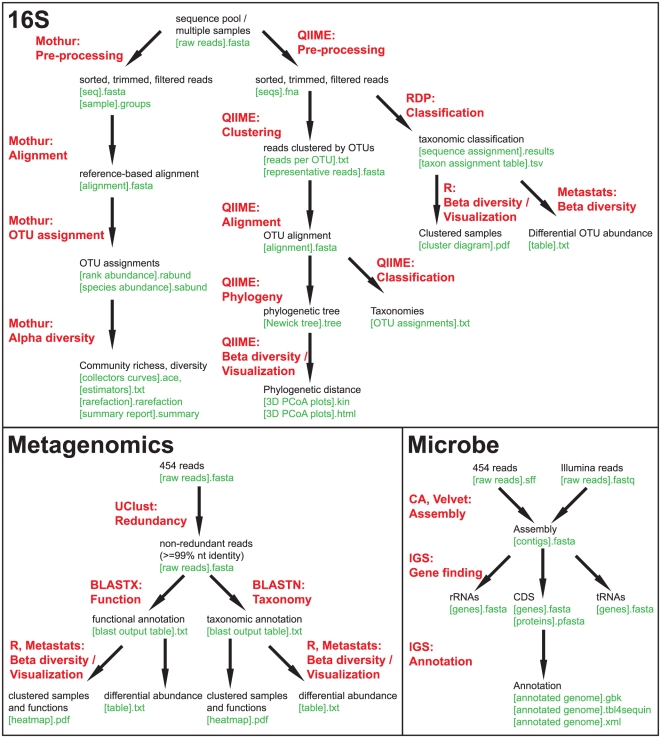
Overview of the CloVR-supported microbial sequence analysis protocols. 1. CloVR-16S supports analysis of pyrotagged amplicon pool sequence data as well as individual samples sequence data, using components from the Mothur [21] package for preprocessing, alignment, operational taxonomic unit (OTU) assignment and alpha diversity estimation. QIIME [22] components are used for sequence clustering, alignment, phylogenetic inference and beta diversity estimation. Sequence reads are assigned to taxonomies using the RDP classifier [23]. Additional visualizations are generated with R script implemented in CloVR. Differentially abundant taxa determined with Metastats [24]. 2. CloVR-Metagenomics supports functional and taxonomic assignments of non-redundant whole-genome shotgun (WGS) sequence data from metagenomic samples. Reads are classified based on BLASTX and BLASTN searches against functional (COG [26], optionally KEGG [46], eggNOG [47]) and taxonomic (RefSeq [25]) reference databases, respectively. The results are statistically evaluated using Metastats and visualized using R scripts implemented in CloVR. 3. CloVR-Microbe supports microbial whole-genome sequencing projects, including Illumina and 454 or Sanger sequence assembly with Velvet [29] and Celera assembler (CA) [28], respectively. Gene predictions and annotations are performed using the complex IGS standard operating procedure for automated prokaryotic annotation (IGS) [27].

Four analysis protocols (CloVR pipelines) were utilized in this study, including (i) a parallelized BLAST [20] search protocol (CloVR-Search 1.0); (ii) a comparative 16S rRNA sequence analysis pipeline (CloVR-16S 1.0) [17]; (iii) a comparative metagenomic sequence analysis pipeline (CloVR-Metagenomics 1.0) [18]; and (iv) a single microbial genome assembly and annotation pipeline (CloVR-Microbe 1.0) [19]. Figure 1 gives an overview of the processes involved in the CloVR-16S, CloVR-Metagenomics and CloVR-Microbe pipelines. Detailed pipeline descriptions, including pipeline version numbers, lists of programs used by the pipeline program version numbers and applied options if different from the defaults, can be found in the supplementary material (Text S1).

The 16S rRNA protocol allows for intra- and inter-group comparative analysis (α- and β-diversity), and is based on methods from Mothur [21], Qiime [22], the RDP Bayesian classifier [23], and Metastats [24]. CloVR-16S calculates the number of non-redundant sequences within the total dataset and uses a threshold of 50,000 above which the computationally expensive distance matrix calculation, which is part of the Mothur component of the pipeline, is not performed. The metagenomics protocol performs clustering of redundant sequences, a BLAST-based taxonomic assignment against the NCBI microbial genome Reference Sequence collection (RefSeq) [25] (BLASTN) and a functional assignment against the Clusters of Orthologous Genes (COGs) [26] databases (BLASTX) and further allows for comparative composition analyses between different sequence datasets, using Metastats. The single microbial genome analysis protocol is based on the IGS Annotation Engine [27] (http://ae.igs.umaryland.edu), with the addition that sequence assembly is performed using Celera Assembler [28] for Roche/454 and Sanger platforms-derived sequence data and Velvet [29] for Illumina platform-derived sequence data. This protocol performs a comprehensive annotation including CDS prediction with Glimmer3 [30], ribosomal RNA (rRNA) gene identification with RNAmmer [31], transfer RNA (tRNA) gene identification with tRNAscan-SE [32], and two types of homology searches using BLASTX against UniRef100 and HMMER [33] against the Pfam [34] and TIGRFAM [35] domain databases.

### Pipeline execution

All analyses to evaluate the resources and costs associated with several typical analysis protocols in microbial genomics were performed using the CloVR VM version beta-0.5 (build clovr-standard-2011-12-04-22-00-04) downloaded from the CloVR project website (http://clovr.org). The technical details of the CloVR VM implementation are described in detail in a separate publication [16]. Briefly, CloVR is a VM image based on Ubuntu Linux 10.10 that runs on a local computer and optionally utilizes the cloud, where a distributed architecture allows for high-throughput parallel processing using multiple CPUs. Pipelines are executed using the Ergatis workflow system [36] and utilize Sun Grid Engine (http://wikis.sun.com/display/GridEngine/Home) for job scheduling.

### Computational resources

The local computer used for evaluation was a 64-bit quad core (Intel Xeon E5520 2.27 GHz CPU) with 4 gigabytes of RAM. For local execution, CloVR was run using VMware Player version 2.0.5 build-109488 (http://vmware.com) configured to use a single CPU core and 2012 MB of memory. Amazon EC2 provides numerous instance types with varying CPU speeds, available RAM and storage (http://aws.amazon.com/ec2/#instance). Previous work in [14] showed the choice of c1.xlarge to be most cost efficient amongst the choices for applications such as BLAST. The c1.xlarge instances provide 8 virtual CPU cores, 8 GB RAM per instance, and 400 GB of local temporary disk storage. In this study, each pipeline was run on a separate cluster of instances within the cloud consisting of one master instance and zero or more worker instances. All master instances utilized the c1.xlarge instance type, except for CloVR-Microbe runs on Illumina sequence data that utilized m2.2×large and m2.xlarge instance types. All worker instances utilized c1.xlarge instances, which at the time of preparing this manuscript were priced at $0.68 per CPU hour (CPU hr). Assembly and annotation of Illumina sequence data required master instances with RAM in excess of the c1.xlarge instance capacity. In the case of the assembly and annotation run on single-read Illumina sequence data, the corresponding master instance was an m2.2×large instance ($1.00/CPU hr), while for the paired-end Illumina run we requested an m2.xlarge master Instance ($0.50/CPU hr). Associated pipeline costs on Amazon EC2 were calculated using cluster performance charts, visualized with the Ganglia tool (http://ganglia.sourceforge.net/), which describe the number of instances utilized in each cluster over time. Pipeline runtimes were obtained from the Ergatis workflow system [36].

### Spot market bid-price simulations

To simulate runtime distributions within the Amazon EC2 spot market, we first collected corresponding hourly spot prices for the c1.xlarge instance type from October 20, 2010 to January 24, 2011. Assuming a hypothetical pipeline runtime of 120 CPU hours and a range of bid prices ($0.27/CPU hr to $0.80/CPU hr), we simulated the actual (wall-clock) runtime of a pipeline from random starting points in the collected spot market price data. Given a bid price *β* and a CPU hr requirement *γ*, 500 random starting points were picked between October 20, 2010 and January 24, 2011, and the runtime was calculated assuming no processes were running whenever the spot price was above the bid price *β*. For example, if the bid price was constantly greater than or equal to the spot price, the actual runtime would be *γ*, because the requested price was always met. Alternatively, if the bid price fell below the spot price for a single hour, then no work was done in that hour and the total actual runtime was *γ* +1. In these simulations, if a simulated pipeline extended beyond January 24, 2011, the remaining runtime was calculated as continued from the beginning of the time-series. Runtime distributions were visualized in R (http://www.r-project.org/). To support the dynamic nature of the spot market, CloVR utilizes a workflow system that supports resuming pipelines from point of failure [36], allowing for reprocessing of work units that fail on hosts that are terminated due to rising spot market prices.

## Results

### Computational requirements of microbial genomics applications

Representative datasets from two next-generation sequencing platforms, the Roche/454 GS (GS FLX and GS FLX Titanium) and Illumina GAIIx (Table 1), were processed with several pipelines for microbial sequence analysis (CloVR-16S, -Microbe, -Metagenomics, and -Search) (Fig. 1, see also Tables S1, S2, S3) to determine processing requirements for typical microbial genome projects (Table 2). This data provides guidelines that can help identify applications that are amenable to execution on a local computer and determine those that benefit particularly from additional resources of the cloud. The datasets evaluated include typical outputs of single or multiple sequencing reactions of the Roche/454 and Illumina platforms or fractions thereof and stem from published data from sequencing projects that received wide recognition in the microbial genomics field [37]–[42] as well as unpublished data from ongoing sequencing projects at the Institute for Genome Sciences (Table 1).

**Table 1:** Example datasets used for CloVR pipeline benchmarking.

Dataset	Data type	Sequencing platform	Library type^1^	Total reads	Units^2^	Avg. read length [bp]	Size [MB]	Samples
**CloVR-Search**								
Infant gut WGS [38]	WGS	454 Titanium	SE	595816	0.6 plates	244	145.3	12
Metahit 500 K [39]	WGS	Illumina GAII	-	500000^3^	1/80 channels	75	37.5	1
**CloVR-16S**								
Humanized mice [41] 4	Amplicon	454 GS FLX	SE	530030	1.1 plates	232	122.5	215
Infant gut 16S [38]	Amplicon	454 GS FLX	SE	399127	0.8 plates	179	95.1	63
Human vagina [40]	Amplicon	454 GS FLX	SE	901264	1.8 plates	223	200.6	392
**CloVR-Metagenomics**								
Obese twins [42]	WGS	454 GS FLX	SE	999990	2 plates	219	218.9	18
Infant gut WGS^4^	WGS	454 Titanium	SE	595816	0.6 plates	244	145.3	12
Nine biomes [37]	WGS	454 GS FLX	SE	5785371	11.6 plates	109	631.2	45
**CloVR-Microbe**								
*Escherichia coli* 250 K	WGS	454 Titanium	PE (3 kbp)	250000^3^	0.25 plates	279	69.7	1
*Escherichia coli* 500 K^4^	WGS	454 Titanium	PE (8 kbp)	500000^3^	0.5 plates	367	183.9	1
*Escherichia coli* 8 M SE	WGS	Illumina GAII	SE	8000000^3^	0.2 channels	36	288	1
*Escherichia coli* 8 M PE	WGS	Illumina GAII	PE (3 kbp)	8000000^3^	0.2 channels	49	392	1
*Acinetobacter baylyi* 250 K	WGS	454 Titanium	PE (8 kbp)	250000^3^	0.25 plates	338	84.7	1

1 Abbreviations: bp, basepairs; SE, single-end; PE, paired-end (in parentheses: insert size); WGS, whole-genome shotgun.

2 References for unit sizes: Roche/454 GS GS FLX, 500 K reads per plate (two half plates); Roche/454 GS GS FLX Titanium, 1 M reads per plate (two half plates); Illumina GAII, 40 M reads per channel (eight channels per flowcell).

3 Trimmed datasets.

**Figure 2:**
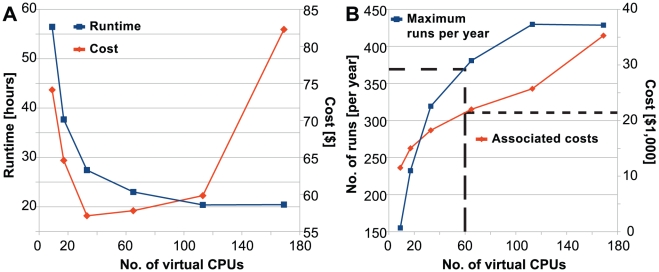
Cost and performance of CloVR-Microbe using different cluster sizes. A) Steps of the CloVR-Microbe pipeline can be executed in parallel to improve performance as shown by plotting pipeline runtimes (blue) and associated costs (red) against the number of CPUs used to perform the analysis on Amazon EC2. B) Using this data, the theoretical maximum throughput per year (blue) as well as associated costs (red) of analysis can be extrapolated. As an example, the output of a single 454 GS FLX Titanium machine, run every other day with two single microbial genomes per sequencing plate (365 total runs), can be processed on Amazon EC2 using 60 CPUs (or eight Amazon EC2 c1.xlarge instances) for less than $25,000, as indicated by the dashed red and blue lines. Inefficiencies in pipeline implementation resulted in increased competition for resources, longer runtimes, and thus increased costs for clusters containing 2 and 3 instances (16 and 24 CPUs, respectively).

**Figure 3:**
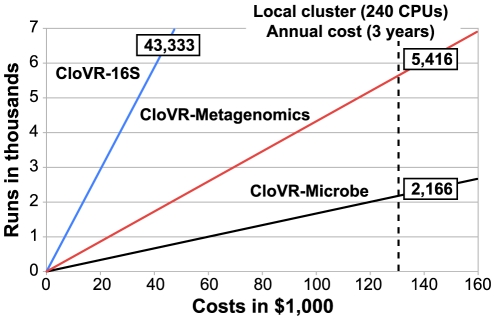
Costs and throughput of CloVR-16S, CloVR-Metagenomics and CloVR-Microbe analysis runs. Costs for single CloVR-16S (blue), CloVR-Metagenomics (red) and CloVR-Microbe (black) runs of comparable datasets (∼500 K 454 GS FLX or GS FLX Titanium reads, see Table 1) on Amazon EC2 were extrapolated to calculate the number of runs that are obtainable for a given dollar value. The black dashed line represents the average annual cost ($130 K) to set up and maintain a local cluster of 240 CPUs for a three years from Dudley *et al*. [15]. Numbers in boxes show how many runs of CloVR-16S, -Metagenomics, and -Microbe can be afforded for the same cost. As an example, approximately three 454 GS FLX Titanium sequencers (two genomes per sequencing plate and one run per day, adding up to 2,190 datasets) or one Illumina GAIIx sequencer (five genomes per lane, eight lanes per sequencing flow cell and one run per week, adding up to 2,080 datasets) can be processed with CloVR-Microbe on Amazon EC2 annually for the same cost as estimated to set up and maintain the 240 CPU local cluster. The local cluster would, however, provide resources exceeding those required for each of the projected analysis protocols.

4 Dataset used for Figures 2 and 3.

**Table 2:** Cost and runtime parameters of CloVR pipeline runs on example datasets.

Dataset	Upload time	Pipeline runtime	Download time	Total cost^1^	Max. VM instances^2^	Max. CPUs		QC	
**CloVR-Search**							**RefSeq matches**		
Infant gut WGS [38], BLASTN against RefSeq	3 min	1 hr 26 min	20 min	$11	8	64	34.3		
Metahit 500 K [39], BLASTX against NR	11 min	10 hr 42 min	17 min	$151	20	160	3.2%		
**CloVR-16S**							**OTUs**		
Humanized mice [41]	42 min	1 hr 30 min	12 min	$3	1	8	14363		
Infant gut 16S [38]	3 min	42 min	10 min	$1	1	8	3447		
Human vagina [40]	1 hr 17 min	1 hr 51 min	14 min	$3	1	8	4967		
**CloVR-Metagenomics** 3							**nr reads**	**RefSeq matches**	**COG matches**
Obese twins [42]	8 min	2 hr 25 min	24 min	$30	20	160	93.6%	33.3%	29.6%
Infant gut WGS	7 min	2 hr 17 min	29 min	$24	15	120	98.2%	35.2%^4^	33.5%
Nine biomes [37]	15 min	5 hr 35 min	39 min	$56	20	160	89.9%	9.3%	5.6%
**CloVR-Microbe**							**Scaffolds/Contigs**	**N50** 5	**CDS** 6
*Escherichia coli* 250 K	24 min	16 hr 21 min	52 min	$55	14	112	8/414	25 kbp	6313
*Escherichia coli* 500 K	20 min	20 hr 23 min	50 min	$60	15	120	37/141	183 kbp	5827
*Escherichia coli* 8 M SE	12 min	15 hr 44 min	37 min	$62	15	120	553/553	17 kbp	4803
*Escherichia coli* 8 M PE	16 min	15 hr 2 min	44 min	$44	15	120	481/481	18 kbp	4464
*Acinetobacter baylyi* 250 K	20 min	9 hr 46 min	37 min	$39	15	120	4/38	262 kbp	3417

1 Rounded to the next full dollar.

2 VM instances are linked together as a cluster for parallel processing on the cloud. The number of instances in a cluster can change during pipeline execution. The master instance is included.

3 The standard CloVR-Metagenomics pipeline refers to the gene prediction-independent protocol.

4 For the CloVR-Metagenomics pipeline sequence reads are clustered, representative reads for each cluster searched against the reference database, and matches of the representative reads assigned back to all reads from the cluster, resulting in a slightly larger number of overall matches than for the comparable CloVR-Search pipeline.

5 Scaffold or contig N50 is a weighted median statistic such that 50% of the entire assembly is contained in scaffolds or contigs equal to or larger than this value.

6 CDS, coding sequences.

CloVR-16S was always run on a single CPU, either on a local desktop or on the c1.xlarge instance of the Amazon EC2. All runs finished in less than three hours (see Table S1 for a comparison of local and EC2-based runs). Processed datasets included up to ∼900 K Roche/454 GS FLX reads from ∼400 samples. Besides the dataset size, runtimes were mostly affected by the species diversity within the dataset. The 530 K humanized mouse gut sequences from 215 different samples [41], for example, which contain a total of 14,363 operational taxonomic units (OTUs), were processed in about the same time as the 901 K human vaginal sequences from 392 samples [40], which only contain 4,967 OTUs.

CloVR-Microbe and CloVR-Metagenomics analyses of all datasets were performed exclusively on Amazon EC2 where all runs finished in less than 24 hours (Table 2). Dataset sizes for CloVR-Metagenomics ranged from ∼600 K reads (454 GS FLX Titanium), corresponding to 1.2 full sequencing plates, to 5.8 million reads (454 GS FLX), corresponding to 11.6 full sequencing plates, all of which were processed in less than six hours on Amazon EC2. Additional time due to upload of input and download of output was consistently less than one hour. Input data sizes for CloVR-Microbe were representative of typical microbial genome project work loads and included sequence read numbers corresponding to a quarter (250 K) or a half (500 K) plate of 454 GS FLX Titanium and 1/5 (8 million) of an Illumina GAIIx lane (single read and paired-end read libraries). Pipeline outputs were found to be in agreement with results from previously processed similar projects in terms of number of detected OTUs, relative OTU compositions, principal coordinate analysis plots of OTU assignments (CloVR-16S), number of functionally and taxonomically assigned reads (CloVR-Metagenomics), number and lengths of contigs, number and functional annotation of genes (CloVR-Microbe). Cluster sizes on Amazon EC2 were configured automatically based on input data sizes using BLAST and other runtime predictions as implemented in CloVR [16]. The estimates for our evaluation ranged from 14 to 15 machine instances, comprising up to 160 virtual CPUs (Table 2).

BLASTN searches of metagenomic WGS sequence data against the NCBI RefSeq collection were performed on Amazon EC2 using CloVR-Search. Using the multi-CPU support of Amazon EC2, ∼600 K reads of 454 GS FLX Titanium, corresponding to 0.6 full plates could be processed in less than two hours (64 CPUs maximum usage). In comparison, a BLASTX search of a similar number (500 K) of shorter (75 bp) Illumina GAIIx reads against the non-redundant protein database at NCBI (NCBI-NR comprising 14.7 M sequences, ∼5000 M residues), which produced about the same percentage of matches (3.2% vs. 3.4%) took about 10 times longer to complete (∼11 hours), using 2.5 times the amount of CPUs (160 CPUs maximum usage). For the Illumina GAIIx platform, 500 K reads correspond to only 1/6 of the average sequencing output of a single channel (eight lanes per flow cell).

The impact of different analysis protocols on runtime and cost for metagenomic WGS analysis was determined by comparing an assembly-based (CloVR-Microbe), a gene-prediction-based and a gene prediction-independent (CloVR-Metagenomics) protocol and a BLAST search (CloVR-Search) on the same dataset (Table S2).

### Real dollar values of bioinformatics sequence analysis applications

Real dollar costs were calculated for all microbial sequence analyses performed with the CloVR protocols on Amazon EC2, in order to provide guidelines for costs associated with microbial genomics projects (Table 2). The costs include overhead introduced by the CloVR VM to make use of the cloud environment, e.g. time for data upload and download and to prepare input and output data. Table 2 also provides example network transfer times for upload to and download from Amazon EC2, although such times can vary substantially based on the network environment. Several large datasets that are used as reference data for the CloVR pipelines, e.g. the UniRef100 protein database for CloVR-Microbe comprising 3.4 GB of compressed data, were hosted permanently on the Amazon Simple Storage Service (http://aws.amazon.com/s3/). This service provides data storage inside the cloud network and was used to reduce the need for data transfer over the Internet when executing in the cloud. During the pipeline execution, the free ephemeral instance storage was used as temporary storage and all output data was compressed and downloaded to the local desktop upon pipeline completion. CloVR is configured to automatically shut down all CloVR VMs on the cloud upon pipeline completion in order to avoid charges for idle instances and persisting storage.

Based on the CloVR runs on Amazon EC2, the cost of each 16S rRNA community analysis was less than $10. For the sequence data generated with the short amplicon 454 sequencing protocol, costs ranged from less than $1 to $2.72. Since all pipelines finished in less than two hours, the costs associated with Amazon EC2 charges for instances being active during upload and download times constitute a significant fraction of the total cost (Table 2), but are nominally small at $0.68 per EC2 c1.xlarge instance hour.

All CloVR-Metagenomics and CloVR-Microbe runs were completed at costs of less than $100. Sequence analyses with the CloVR-Metagenomics pipeline had an associated cost of between ∼$23 and ∼$56; CloVR-Microbe runs had costs of between ∼$39 and ∼$62.

### Capacity and optimization of processing pipelines

The multi-CPU capabilities of the cloud allow for decreased runtime for pipelines involving analysis steps that can be parallelized, such as CloVR-Microbe and CloVR-Metagenomics, which contain BLASTX and BLASTN sequence comparisons (Fig. 1). At the same time, partitioning the analysis with CloVR into multiple parallel processes on different CPUs of the same instance or even across different instances of the same cluster involves copying of reference data, increases the amount of data transfer between instances and incurs additional processing overhead [16]. To determine differences in the CloVR-Microbe runtimes and associated costs depending on the number of CPUs used, the same 454 GS FLX Titanium dataset, 500 K reads corresponding to one full plate of 8 kbp paired-end sequences, was run with different cluster sizes on Amazon EC2 (Fig. 2, Table S3). Based on this example, the lowest runtimes and costs achieved fell between 72 CPUs (23 hours, $58) and 120 CPUs (20 hours, $60). These numbers represent a runtime and cost improvement of up to 36 hours and $16 compared to a 56 hour-run with 16 CPUs for $74. A further increase of the cluster size to 172 CPUs did not result in runtime improvements but resulted in increased cost ($82) due to payment for under-utilized instances. Inefficiencies in pipeline implementation resulted in increased competition for resources, longer runtimes, and thus increased costs for clusters containing two and three instances (16 and 24 CPUs, respectively). Future work on optimizing the CloVR pipelines is expected to reduce runtimes and costs on smaller clusters. A local run on a single-CPU machine was canceled after 14 days and was extrapolated to require in excess of 24 days runtime.

To estimate the amount of sequence analysis that is affordable for a given dollar value, the number of analysis runs using three different protocols (CloVR-16S, CloVR-Metagenomics and CloVR-Microbe) was plotted against the corresponding cost, using results from Table 2 (Fig. 3). These costs were compared to the $130 K estimated as average annual cost to set up and maintain a local cluster of 240 CPUs for three years as described by Dudley *et al*. [15]. Using the Dudley estimates, for the cost of a local cluster, 43,333 runs of CloVR-16S; 5,416 runs of CloVR-Metagenomics; and 2,166 runs of CloVR-Microbe can be processed each year on Amazon EC2. For single whole-genome microbial sequencing projects, with a theoretical annual output of 730 datasets per 454 GS FLX Titanium sequencer (one full plate per day, two single-genome datasets per plate), up to three sequencing machines can be supported using Amazon EC2 at current prices, using CloVR-Microbe benchmark protocols, before the estimated cost of a local cluster is reached. It should be noted that the interpretation of these results is limited by the fact that the comparison does not consider utilization rates on the local cluster, which is likely to be used for a different applications, rather than to exclusively support a single protocol.

### Realizing cost savings using excess capacity in the Amazon EC2 spot market

The Amazon EC2 spot market allows customers to place bids on unused cloud resources and utilize instances for as long as the bid exceeds the current spot price (http://aws.amazon.com/ec2/spot-instances/). During periods of weak demand, the spot market provides the ability to utilize excess resources at a discounted price. Over the period of the past year, the spot market price for the c1.xlarge instance averaged $0.26 compared to an on-demand price of $0.68. This variable pricing is well-suited to processing needs that are not time critical, since analyses purchased under this model will only proceed when the provided bid price is above the current market price for the resource. This market model also provides the ability to predict the expected completion time of a pipeline for a particular bid price using historical pricing data.

**Figure 4:**
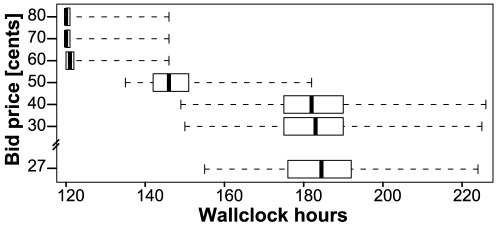
Predicted runtimes using varying bid prices for the Amazon EC2 spot market. An analysis requiring 120 CPU hours was used an example to estimate the expected completion time for different bid prices for the Amazon EC2 c1.xlarge instance, ranging from $0.27 to $0.80 (on-demand price: $0,68).

To evaluate potential cost savings and associated runtime increases that could be achieved with the Amazon EC2 spot market, the expected completion times were estimated for bids of $0.27 to $0.80 using a hypothetical analysis requiring 120 c1.xlarge instance hours (960 c1.xlarge CPU hours) for completion (Fig. 4). The expected completion time was predicted for each bid price using the recorded pricing data for the past month. Based on this model, at a bid price of $0.68 the analysis was expected to execute in ∼120 hours, while never taking longer than ∼145 hours. By comparison, a $0.27 bid, which during the recorded month was not fulfilled during times of peak demand, when the market price rose above the bid, realized savings of 40% for the user. A bid of $0.27 was estimated to result in an average runtime of ∼185 hours, 50% more than when using the full on-demand price. Altogether, predicted runtimes for this bid ranged from ∼155 hours (29% slower) to ∼225 hours (87% slower). In this example, runtimes were estimated based on a task that was executed on a single CPU, whereas many bioinformatics pipelines utilize multiple CPUs in parallel across several instances, thereby reducing the actual pipeline runtime.

## Discussion

In this study, we explore the costs and resources required for microbial sequence analysis using pre-packaged protocols in CloVR [16]. By packaging these pipelines into a single automated framework, the CloVR virtual machine, the performance of protocols between platforms and costs on commercial clouds can easily be compared and evaluated.

The automated pipelines in CloVR were selected with the intention of packaging existing community-supported analysis protocols. The protocol, CloVR-Microbe, combines a sequence assembly step with the IGS Annotation Engine [27]. With the support of a large local grid cluster, the IGS Annotation Engine was designed to be thorough for genome annotation but not optimized for speed or efficient CPU usage, and many alternative genome annotation protocols exist, e.g. RAST [43], DIYA [44]. To our knowledge, CloVR-Microbe represents the first automated pipeline that combines sequence assembly and annotation in an automated pipeline that runs on the desktop.

The CloVR-16S pipeline was designed to combine components of several widely used 16S rRNA sequence analysis protocols, without making the entire workflow computationally too complex to process even large sequence datasets (>200 samples, >500 K sequences). The current implementation of CloVR-16S supports a distance matrix-based operational taxonomic unit (OTU) assignment and α-diversity analysis with Mothur [21], direct taxonomic classifications of sequence reads with the RDP classifier tool [23] and microbial community analysis with the QIIME tool, which has a strong focus on tree-based metric for β-diversity analysis [22]. A critical component of CloVR-16S in its current implementation is the threshold of 50,000 non-redundant sequences above which the Mothur component with its computationally expensive distance matrix calculation is not carried out.

Metagenomics projects are usually designed to generate the most sequence data per invested dollar and, thus, often involve large-scale next-generation sequencing. Due to the resulting dataset sizes, metagenomics analysis protocols often rely on the direct classification of individual sequence reads by BLAST, instead of using sequence assembly, which is computationally demanding. Similarly, the CloVR-Metagenomics pipeline was built to examine and compare taxonomic and functional microbial community compositions within and between metagenomic samples using two BLAST searches against a bacterial genome database (BLASTN against NCBI's RefSeq) and against a functionally annotated protein database (BLASTP against NCBI's COG). The CloVR-Search pipeline was designed to provide support for large-scale BLAST comparisons using the cloud multi-processor architecture. A direct BLASTN comparison of each sequence read against the NCBI RefSeq nucleotide database with CloVR-Search was shown to provide runtime improvements albeit without producing the visual and statistical evaluations of the results that are generated by the CloVR-Metagenomics pipeline. BLAST search results alone can be used in downstream applications, e.g. with the MEGAN tool, which utilizes pre-computed BLAST result to calculate taxonomic classifications of metagenomic sequence data [45].

The comparison of two pipelines for processing metagenomics WGS data demonstrates that the choice of analysis protocols is most critical and will be the primary determinant of performance rather than the execution environment. Runtimes and costs for the same dataset differed significantly, depending on whether the data was processed using assembly-based, gene prediction-based or gene prediction-independent protocols (Table S2). A recent publication suggests that for the analysis of metagenomics WGS data, an exhaustive translated BLASTX against NCBI-NR may be prohibitively time-consuming and expensive on the cloud [14]. Similarly, based on our calculations, the BLASTX protocol is expensive for searching Illumina short read data (11 hours, $150 for 500 K reads), such as those generated from the recent MetaHIT study on the human gut microbiota [39]. In summary, these results support the notion that the bioinformatics bottleneck of next-generation sequence data will not be completely addressed simply by scaling up the computational resources without utilization of methods specifically designed for large data volumes.

Cloud computing has caused notable excitement in the bioinformatics community as a potential solution to the so-called “bioinformatics bottleneck”, resulting from the increasing production of second- and third-generation sequence data with high computational demands for analysis [7], [8]. There is, however, concern and uncertainty over the costs of using commercial cloud resources. We decided to use the popular Amazon EC2 cloud as a model for evaluating analysis costs. Importantly for budgeting, the costs at Amazon EC2 are transparent and directly obtainable for any workload, allowing for attaching real dollar costs to computational analyses. Our results show that bioinformatics support for microbial genomics can be provided at a competitive price, provided analysis protocols are chosen carefully. In addition, as many analysis needs are not time-critical and can wait for off-peak hours, a bidding market for computational resources, such as the Amazon EC2 Spot Market, provides an intriguing model for further cost savings. Since these costs depend substantially on the choice of analysis protocol, the results in this study can also be used as benchmarks for comparing costs and resources of other analysis protocols.

The Amazon EC2 cloud can also serve as a model to evaluate the computational infrastructure needed to perform common microbial genomics applications. Our tests with the CloVR protocols show that typical workloads of small to midsize sequencing facilities are economically processed either locally, on a single desktop machine (CloVR-16S), or online using the Amazon EC2 cloud (CloVR-Metagenomics, -Microbe, -Search). The computational resources deployed for the evaluation were modest, utilizing no more than 20 virtual machine instances, eight CPUs per instance and 152 CPUs at a maximum, indicating that use of comparable resources through shared local computing infrastructures is also feasible. As multi-core CPUs are increasingly becoming accessible on the desktop computer market, the ability to process larger data on local desktops is also likely to increase in the future.

Our evaluation datasets were small (631 MB maximum) and network transfers were not prohibitively long (<1.5 hours maximum), although we do expect that network transfer can contribute significantly to overall runtime for larger datasets or lower speed connections. Although raw data output sizes may increase from new sequencing platforms, in the case of single-genome projects, the amount of raw sequence data necessary for whole genome assembly and annotation is not expected to grow dramatically in the near future. The overhead costs resulting from data transfers, either because Amazon EC2 charges directly for the transfer itself ($0.1 per GB of inbound and $0.15 per GB of outbound data transfer, April 2011) or for the instance, which is online during the transfer, are insignificant in our evaluations, totaling no more than a few dollars. It is noteworthy that Amazon allows for data import using physical storage devices sent to the Amazon Web Services (http://aws.amazon.com/importexport/).

In a recent publication the Amazon EC2 cloud was reviewed favorably as an alternative to local compute clusters for large-scale microarray data analysis [15]. In the same publication the cost to set up a local compute cluster (240 CPUs) was estimated as about $130 K per year (for three years total). Using our example calculations and an average of two datasets per 454 GS FLX Titanium sequencing run (500 K reads each) 59, 7 and 3 454 GS FLX Titanium sequencers running daily could be supported with the CloVR-16S, CloVR-Metagenomics and CloVR-Microbe protocols, respectively. However, it should be noted that the capacities of the local compute cluster used for the comparison would exceed those required for the analysis examples. The excess in computational capacities provided by the local cluster would allow for resource sharing with additional users to reduce overall costs or provide computational support for bioinformatics research and development, which can be critical especially in the academic environment.

In general, local cluster setups and cloud computing services are best compared when taking into account average utilization rates and expectations on process runtimes. The on-demand model of the cloud makes it most attractive when compared to a local cluster that is under-utilized since paying for idle cycles is avoided. Local clusters, on the other side, can be better designed to meet the exact requirements for particular analysis types, for example by providing high memory machines or fast networking, which may be unavailable on the cloud. In academic or other research settings, cost savings for local setups are realized by integrating the local compute cluster into a core facility, allowing for multiple user support, shared expenses for maintenance, and operation at levels closer to maximum capacities. In cases where a local resource achieves a very high utilization rate, the benefits and cost savings of an on-demand model may disappear. However, there is a considerable challenge in right-sizing the resource to both achieve a high utilization rate and deliver results in a reasonable amount of time.

The microbial genomics field is in the middle of experiencing a fundamental re-organization, as sequence generation is increasingly becoming decentralized and introduced as a standard application not only in smaller research laboratories but also the clinical and public health sector. This development requires a concomitant decentralization of the sequencing-associated bioinformatics, i.e. widespread access to bioinformatics expertise and computing infrastructures, as well as improved transparency of associated cost and required infrastructures. The CloVR project aims at closing the bioinformatics gap by providing automated pipelines and support for cloud computing from the local desktop (http://clovr.org). The results presented, which use CloVR in combination with the Amazon cloud, attach transparent cost and runtime calculations to common microbial genomics applications. All benchmarks provided here are specifically tied to the CloVR analysis protocols, CloVR implementation, Amazon cloud hardware, and size of data sets, and changes in any of these areas may alter the benchmarks. Users should therefore consider carefully if the examples provided will apply to a particular sequence analysis task. However, the results presented here show that microbial sequence analysis is generally affordable to the broad user community and that cloud computing provides an economical resource for microbial genomics analysis pipelines, such as those implemented in CloVR. As virtualization and cloud computing have found widespread applications in sequence analysis, we expect the ability to evaluate and compare the cost and scalability of bioinformatics applications will increase in the future.

## Supporting Information

Text S1 **Detailed descriptions, including pipeline version numbers, lists of programs used by the pipeline program version numbers and applied options if different from the defaults for CloVR-16S v1.0, CloVR-Metagenomics v1.0 and CloVR-Microbe v1.0.**
(PDF)

Table S1 **Comparison of CloVR-16S runtimes executed locally and on Amazon EC2.**
(XLSX)

Table S2 **Variations in cost and runtime parameters of different CloVR pipeline runs on the same metagenomics WGS dataset (Infant gut WGS).** Three different analysis protocols (CloVR-Microbe, CloVR-Metagenomics and CloVR-Search) were evaluated for their impact on runtime and cost for metagenomic WGS analysis. All analyses were run on the Infant Gut Microbiome WGS input dataset [38], corresponding to 0.6 full plates of single-end 454 GS FLX Titanium sequences. The CloVR-Microbe pipeline was included to provide a comparison of assembly-based and assembly-free analysis methods. We note that the Glimmer gene finding tool [30], which is part of the CloVR-Microbe protocol, was optimized for large contiguous assembled sequence data and is known to perform less optimally on short sequence fragments that contain a large number of truncated coding sequences. Two variations were used of the CloVR-Metagenomics protocol: i) The first searches each nucleotide sequence read against the COG database [26] by BLASTX, using all six nucleotide sequence frames translated into protein sequences, whereas ii) the second first runs a gene prediction with Metagene [48], before translating the identified genes into protein sequences and running a BLASTP search against the COG database. A BLASTN comparison of each read against NCBI's RefSeq database performed with CloVR-Search was used as the most basic analysis protocol.
(XLSX)

Table S3 **Runtime and cost comparisons of CloVR-Microbe executions on the same input dataset run with different Amazon EC2 cluster sizes.**
(XLSX)
