# Supplementary material for: Resources and Costs for Microbial Sequence Analysis Evaluated Using Virtual Machines and Cloud Computing
Source: PLoS One. 2011 Oct 19;6(10):e26624. doi: 10.1371/journal.pone.0026624 (PMC3197577; doi:10.1371/journal.pone.0026624)
Supplement: Text S1 — Detailed descriptions, including pipeline version numbers, lists of programs used by the pipeline program version numbers and applied options if different from the defaults for CloVR-16S v1.0, CloVR-Metagenomics v1.0 and CloVR-Microbe v1.0. (PDF) [file pone.0026624.s001.pdf]

CloVR-16S: Phylogenetic microbial community composition analysis based on 16S ribosomal RNA amplicon sequencing – standard operating procedure, version1.0

James Robert White, Cesar Arze, Malcolm Matalka, the CloVR team, Samuel V. Angiuoli & W. Florian Fricke

The Institute for Genome Sciences, University of Maryland School of Medicine, Baltimore, MD 21201, USA

Abstract
The CloVR-16S pipeline employs several well-known phylogenetic tools and protocols for the analysis of 16S rRNA sequence datasets:

A) Mothur [1] – a C++-based software package used for clustering 16S rRNA sequences into operational taxonomic units (OTUs). Mothur creates OTUs using a matrix that describes pairwise distances between representative aligned sequences and subsequently estimates within-sample diversity (alpha diversity);
B) The Ribosomal Database (RDP) naive Bayesian classifier [2] assigns each 16S sequence to a reference taxonomy with associated empirical probabilities based on oligonucleotide frequencies;
C) Qiime [3] – a python-based workflow package, allowing for sequence processing and phylogenetic analysis using different methods including phylogenetic distance (UniFrac [4]) for within- (alpha diversity) and between- (beta diversity) sample analysis;
D) Metastats [5] and custom R scripts used to generate additional statistical and graphical evaluations.

Though some of the different protocols used in CloVR-16S overlap in purpose (e.g. OTU clustering), the end-user benefits from their overall complementary nature as they focus on different aspects of the phylogenetic analysis. CloVR-16S accepts as input raw multiplex 454-pyrosequencer output, i.e. pooled pyrotagged sequences from multiple samples, or alternatively, pre-processed sequences from multiple samples in separate files. This protocol became first available in CloVR beta versions 0.5.

Software

Step	Program	Version	Weblink	Reference
Distance-based OTU classification and phylogeny	Mothur	1.12.0	http://www.mothur.org/	[1]
Bayesian taxonomic classification	RDP classifier	2.0	http://rdp.cme.msu.edu/classifier/classifier.jsp	[2]
Phylogenetic distance-based sample comparison and phylogeny	Qiime	1.1.0	http://qiime.sourceforge.net/	[3]
Differential taxonomic prevalence calculation	Metastats	1.0	http://metastats.cbcb.umd.edu/	[5]
Statistical and graphical evaluation	R	2.10.1-2	http://www.r-project.org/	

Reference data

Database	Data	Version	Weblink	Reference
Silva	Curated 16S and 18S rRNA sequence alignment	102	http://www.arb-silva.de/	[6]
Greengenes	Curated 16S rRNA sequence alignment (core set imputed)		http://greengenes.lbl.gov/	[7]
	Lane mask		http://greengenes.lbl.gov/	[8]

Pipeline input

Data	Suffix	Description
Multiple sequence pool	.fasta	Pool of un-trimmed, un-checked, un-binned pyrotagged 454 sequences
Multiple fasta files	.fasta	Trimmed and binned sequences, 1 file per sample
Metadata	.txt	Sample-associated feature table (tab-delimited)

Pipeline output

Data	Suffix	Description
Taxonomic assignments	.tsv	Taxonomic classification of every read (RDP classifier/Qiime)
OTU assignments	.txt	Table showing OTU sample compositions (RDP classifier/Qiime)
	.html	OTU heatmap (Qiime)
Alpha diversity	.txt	Collectors curves (Mothur)
	.txt	Rarefaction curves (Mothur)
	.txt	Richness and diversity estimates (Mothur)
	.txt	Richness and diversity estimates (Qiime)
	.txt	Summary report (Mothur)
Beta diversity	.kin	Weighted and Unweighted UniFrac 3D PCoA plots (Qiime)
	.pdf	Taxonomic composition-based sample clustering (CloVR)
	.pdf	Taxonomic composition-based stacked histograms (R)
	.csv	Differentially abundant taxonomic groups (Metastats)

A. Requirements for pipeline Input

To run the full CloVR-16S analysis track, at least two different input files have to be provided by the user: a sequence file in the FASTA format and a tab-delimited metadata file (.txt). Sequence data may consist of a single .fasta file that contains sequences from multiple samples, individually pyrotagged by sample-specific barcodes as commonly used in the *454 Amplicon Sequencing* protocol (http://www.454.com/products-solutions/experimental-design-options/amplicon-sequencing.asp). No two FASTA headers within any submitted file may be identical. The metadata file provides sample-associated information with the following formatting requirements, based on the Qiime mapping file.

A.1. Metadata file requirements for runs on a single sequence pool

```
#SampleID     BarcodeSequence   LinkerPrimerSequence     Treatment    Description
Sample_1      AGCACGAGCCTA      CATGCTGCCTCCCGTAGGAGT    Control      mouse_ID_300
Sample_2      AGCACGAGCCTA      CATGCTGCCTCCCGTAGGAGT    Diabetic     mouse_ID_354
Sample_3      AACTCGTCGATG      CATGCTGCCTCCCGTAGGAGT    Control      mouse_ID_355
Sample_4      ACAGACCACTCA      CATGCTGCCTCCCGTAGGAGT    Diabetic     mouse_ID_356
```

The following rules apply:

1. All entries are tab-delimited.
2. All entries in every column are defined (no empty fields).
3. The header line begins with the following fields:
"#SampleID<tab>BarcodeSequence<tab> LinkerPrimerSequence".
4. The header line must end with the field "Description".
5. The BarcodeSequence and LinkerPrimerSequences fields have valid IUPAC DNA characters.
6. There are no duplicate header fields.
7. No header fields or corresponding entries contain invalid characters (only alphanumeric and underscore characters allowed).
8. There are no duplicates when the primer and barcodes are appended.

A.2. Metadata file requirements for runs on multiple sequences

Multiple fasta files can be provided so that each file comprises sequences from different samples. In this case, the metadata file must meet the following requirements:

```
#File       SampleName    ph     Description
A.fasta      sampleA     high     control
B.fasta      sampleB     high      sick
C.fasta      sampleC      low     treated
```

where:

1. All entries are tab-delimited.
2. All entries in every column are defined (no empty fields).
3. The header line begins with: "#File<tab>".
4. There are no duplicate header fields or file names.
5. No header fields or corresponding entries contain invalid characters (only alphanumeric and underscores characters allowed).
6. The header line must end with the field "Description".

Pairwise comparisons: To utilize the Metastats statistical methodology, which detects differential abundances of taxa between two sample groups, the associated header field must end with "`_p`", (e.g. "`Treatment_p`", or "`ph_p`"). If a header with the "_p" ending exists, pairwise Metastats calculations will be carried out between all groups specified in the corresponding column (provided that a group contains at least three samples).

B. Sequence Processing and analysis with Mothur

The Mothur component of CloVR-16S follows in large parts the pyrosequencing 16S rRNA sequence analysis example on the Mothur wiki page (http://www.mothur.org/wiki/Costello_stool_analysis). Sequence pools are pre-processed (trimmed, sorted, filtered), aligned against a reference (the curated Silva 16S and 18S rRNA alignment [6]), further processed to remove redundancy and to filter the alignment, used to generate a distance matrix, clustered and assigned to OTUs. Based on the sample OTU classification, the within-sample community composition is analyzed using common richness and diversity estimators as well as collectors and rarefaction curves (α-diversity).

B.1. Sequence pre-processing

To check each read from the sequence pool for quality and to sort sequences based on the sample-specific barcodes, the "trim.seqs" program is used with the following parameters:

- "minlength=100" (minimum sequence length)
- "maxhomop=8" (maximum homopolymer length)
- "maxambig=0" (maximum number of ambiguous base calls)
- "flip=F" (do not use the reverse complement of the sequences -- *reverse complements are considered in the alignment step*).

All length parameters refer to base pairs (bp). This step generates trimmed .fasta and .groups files, which are used in the downstream analysis.

B.2 Alignment

To speed up the downstream analysis and to facilitate the analysis of large data sets, identical sequences, which can constitute a significant fraction of the sequence data are removed, using the "unique.seqs" command. The non-redundant sequence dataset is then aligned against the Greengenes reference core imputed alignment, which is available from the Greengenes website (http://greengenes.lbl.gov/Download/Sequence_Data/Fasta_data_files), using the "align.seqs" command with the default parameters and "flip=t" (if the alignment of a sequence read falls below the default threshold [0.50], the reverse complement is tried). In order to keep only those sequence reads that produce alignments of a minimum length of 50 bp, the "screen.seqs" command is run with the "minlength=50" option. With the "filter.seqs" command in combination with the "vertical=T" option, any column, which only contains gaps, is removed from the alignment. Since the trimming of the alignment has created new duplicate sequences, identical sequences are removed again using the "unique.seqs" command.

B.3. Threshold for the maximum number of processed sequences

Since the following steps of the Mothur pipeline can be computationally very demanding, the threshold for the number of unique sequence reads from the alignment of the previous step is set to 50,000. If the number of sequences exceeds this threshold, the dataset is not further processed with Mothur but instead analyzed through the remaining CloVR-16S components.

B.4. Clustering and OTU assignment

In Mothur, sequence reads are assigned to OTUs based on uncorrected pairwise distances between all aligned sequences. With the "dist.seqs" command a column-

formatted distance matrix is generated, using the "cutoff=0.10" option, which limits the distance matrix to keep only sequence reads with a distance smaller than 0.10 (at least 90% similar). Using the default "furthest neighbor" option, the "cluster" command assigns sequence reads to OTUs based on the distance matrix generated in the previous step.

B.5. Alpha diversity analysis

To perform the alpha diversity analysis, the OTU clustering results are read into Mothur with the "read.otu" command and the "label=unique-0.03-0.05-0.10" option to output all OTU levels using 97%, 95% and 90% similarity thresholds, respectively. The command "collect.single" generates collectors curves that describe how comprehensively a microbial community has been assessed in the sample. This is done by calculating how community richness and diversity change as more individuals from the community are sampled. "collect.single" is performed with the "freq=5" option, which sets the frequency with which the richness and diversity are calculated to every 5 sequences. To generate intra-sample rarefaction curves, applying a re-sampling without replacement approach, the "rarefaction.single" command is used with the same "freq=5" option. Rarefaction curves provide a way of comparing the richness observed in different samples. The "summary.single" command produces a summary file of various richness and diversity estimators for each sample.

C. RDP classification of all sequence reads

The output of the pre-processing step (B.1. Mothur:trim.seqs), i.e. all sorted, trimmed and filtered sequence reads, are classified using the RDP classifier tool [2], as described on the Ribosomal Database Project website (http://rdp.cme.msu.edu/classifier/classifier.jsp). As output, a results file is created, which contains the taxonomic classification of each sequence read from all samples, including a confidence score (up to 1.0) assigned by the RDP classifier. In addition, tab-delimited table files (.tsv) are generated, which show the composition of each sample at different taxonomic levels, including phylum, class, order, family and genus. Assignments with confidence values below 0.8 (80%) are assigned as “unknown” for the generation of the .tsv files.

D. Sequence processing and analysis with Qiime

The Qiime component of CloVR-16S follows the Overview Tutorial on the Qiime website (http://qiime.sourceforge.net/tutorials/tutorial.html). It uses the same unprocessed sequence pools as the Mothur component as input and takes advantage of three Qiime workflow scripts to combine related steps of the analysis pipeline: "pick_otus_through_otu_table.py" is used for sequence clustering, alignment, classification and phylogenetic tree prediction; and "beta_diversity_through_3d_plots.py" is used to calculate β-diversity estimators and to generate 3D Principal Coordinate Analysis (PCoA) plots for the graphical representation of differences in microbial community compositions between samples (beta diversity).

D.1. Sequence pre-processing

To assign multiplex reads from sequence pools to specific samples using sequence barcodes, as well as to remove low-quality reads and to filter reads by length, the "split_libraries.py" script is used. This step removes all reads from the analysis that do

not have the user-specified barcode sequence. The following options are used: "--min-seq-length 100" (sets the minimum sequence length to 100 bp), "--barcode-type variable_length" (disables barcode corrections), and "--max-homopolymer 8" (sets the maximum homopolymer length to 8 bp).

D.2. Sequence clustering, alignment, classification and phylogenetic tree prediction

The "pick_otus_through_otu_table.py" workflow script calls the following Python scripts: 1) "pick_otus.py" is used to cluster reads from all samples into OTUs based on nucleotide sequence identity. The clustering program for this step is "Uclust" [9] and the nucleotide sequence identity threshold for all reads within an OTU is 97%. 2) "assign_taxonomy.py" uses the RDP classifier [2] with a confidence threshold of 0.8 to assign each OTU-representing read to a known taxon based on the pre-built database from the RDP classifier program. A .txt file is created by this script, which shows the most specific classification of each read above the confidence threshold, i.e. the resolution of the classification varies between reads, showing taxonomic lineages of different lengths. 3) "make_otu_table.py" generates an OTU table from the classification results, together with the information about the number of reads that each OTU represents, which specifies the OTU counts that each sample contains for each taxonomic assignment. 4) "align_seqs.py" uses the PyNAST tool [10] to align OTU-representing reads against the Greengenes reference alignment [7]. 5) "filter_alignment.py" uses the Greengenes Lane mask [8] to defines those positions from the alignment that will be ignored when building the phylogenetic tree. 6) "make_phylogeny.py" uses the "FastTree" program [11] to generate a phylogenetic tree in the Newick format.

D.3. Beta diversity sample analysis

The "beta_diversity_through_3d_plots.py" workflow script calls the following Python scripts: 1) "beta_diversity.py" takes the OTU table and phylogenetic tree as input to calculate beta diversity estimators, including phylogenetic distance as measured through weighted and unweighted UniFrac analysis [4], and to generate a distance matrix. 2) "principal_coordinates.py" maps the multidimensional variation between samples from the distance matrix on three principal coordinates. 3) "make_prefs_file.py" sets the parameters for the PCoA display based on the user-provided metadata information. 4) "make_3d_plots.py" generates 3D PCoA plots in the .html and .kin format, which can be opened with a web browser or the free KiNG Display Software, which is available from http://kinemage.biochem.duke.edu/software/king.php.

E. Additional beta diversity analysis using Metastats and the R statistical package

The output from the taxonomic classification of each sequence read from all samples by the RDP classification step (see section C) and of the RDP-based classification of the OTU-representing sequence reads from all samples by "Qiime:assign.taxonomy" is further analyzed and graphically represented using the "Metastats" program [5] and customized scripts in the R programming language.

E.1. Detection of differentially abundant features

The "Metastats" program uses count data from the taxonomic assignment of sequences with the RDP classifier to compare two groups containing at least three samples each in order to detect features with differential abundance in the two groups [5]. The results are

calculated on different taxonomic levels (phylum, class, order, family, genus) and presented as a table in the .txt format, which shows the mean relative abundance of a feature, variance and standard error together with a p value and q value to describe significance of the detected variations. Note Metastats can run analyses of 1 sample vs. 1 sample, or *N* samples vs. *M* samples, where *N* and *M* are greater than 1. It cannot perform a comparison of 1 sample vs. 2 samples (see project website: http://metastats.cbcb.umd.edu/).

E.2. Stacked histogram generation

Custom R scripts are used to normalize taxonomic group counts to relative abundances. Stacked histograms of the relative abundances as well as the absolute abundances are generated in the .pdf format, if there are at most 50 samples and at most 25 taxon groups. Beyond these limits a visualized histogram is not generated.

E.3. Unsupervised sample clustering

Custom R scripts are used to normalize taxon counts and to calculate distance matrices for samples and taxonomic groups, using a Euclidean distance metric. Complete-linkage (furthest neighbor) clustering is employed to create dendrograms of samples and taxa in the .pdf format. The R packages *RColorBrewer* and *gplots* are utilized.

F. References

1. Schloss PD, Westcott SL, Ryabin T, Hall JR, Hartmann M, et al. (2009) Introducing mothur: open-source, platform-independent, community-supported software for describing and comparing microbial communities. Appl Environ Microbiol 75: 7537-7541.

2. Wang Q, Garrity GM, Tiedje JM, Cole JR (2007) Naive Bayesian classifier for rapid assignment of rRNA sequences into the new bacterial taxonomy. Appl Environ Microbiol 73: 5261-5267.

3. Caporaso JG, Kuczynski J, Stombaugh J, Bittinger K, Bushman FD, et al. (2010) QIIME allows analysis of high-throughput community sequencing data. Nat Methods 7: 335-336.

4. Lozupone C, Knight R (2005) UniFrac: a new phylogenetic method for comparing microbial communities. Appl Environ Microbiol 71: 8228-8235.

5. White JR, Nagarajan N, Pop M (2009) Statistical methods for detecting differentially abundant features in clinical metagenomic samples. PLoS Comput Biol 5: e1000352.

6. Pruesse E, Quast C, Knittel K, Fuchs BM, Ludwig W, et al. (2007) SILVA: a comprehensive online resource for quality checked and aligned ribosomal RNA sequence data compatible with ARB. Nucleic Acids Res 35: 7188-7196.

7. DeSantis TZ, Hugenholtz P, Larsen N, Rojas M, Brodie EL, et al. (2006) Greengenes, a chimera-checked 16S rRNA gene database and workbench compatible with ARB. Appl Environ Microbiol 72: 5069-5072.

8. Lane DJ (1991) 16S/23S rRNA sequencing. In: Stackebrandt E, Goodfellow M, editors. Nucleic Acid Techniques in Bacterial Systematics. New York: Wiley. pp. 115-175.

9. Edgar RC (2010) Search and clustering orders of magnitude faster than BLAST. Bioinformatics.

10. Caporaso JG, Bittinger K, Bushman FD, DeSantis TZ, Andersen GL, et al. (2010) PyNAST: a flexible tool for aligning sequences to a template alignment. Bioinformatics 26: 266-267.

11. Price MN, Dehal PS, Arkin AP (2010) FastTree 2--approximately maximum-likelihood trees for large alignments. PLoS One 5: e9490.

ClοVR-Metagenomics: Functional and taxonomic microbial community characterization from metagenomic whole-genome shotgun (WGS) sequences – standard operating procedure, version 1.0

James Robert White, Cesar Arze, Malcolm Matalka, the CloVR team, Samuel V. Angiuoli & W. Florian Fricke

The Institute for Genome Sciences, University of Maryland School of Medicine, Baltimore, MD 21201, USA

Abstract
The CloVR-Metagenomics pipeline employs several well-known tools and protocols for the analysis of metagenomic whole-genome shotgun (WGS) sequence datasets:

A) UCLUST [1] – a C++-based software package for clustering redundant DNA sequences and removing artificial 454 replicates [1];
B) BLASTX and BLASTN [2] for functional and taxonomic assignment of sequences, respectively;
C) Metastats [3] and custom R scripts to generate additional statistical and graphical evaluation.

The CloVR-Metagenomics pipeline accepts as input multiple fasta files (1 sample per file) and a corresponding tab-delimited metadata file that specifies features associated with the samples, which are used for comparative analysis. This protocol became first available in CloVR beta version 0.5.

Software

Step	Program	Version	Weblink	Reference
Clustering of redundant sequences (replicate removal)	UCLUST	1.1.579q	http://www.drive5.com/usearch	[1]
Functional classification of DNA sequences	BLASTX	2.2.21	http://blast.ncbi.nlm.nih.gov	[2]
Taxonomic classification of DNA sequences	BLASTN	2.2.21	http://blast.ncbi.nlm.nih.gov	[2]
Differential abundance detection	Metastats	1.0	http://metastats.cbcb.umd.edu/	[3]
Statistical evaluation	R	2.10.1-2	http://www.r-project.org/	

Reference data

Database	Data	Versio n	Weblink	Reference
COG	Functionally annotated protein sequences (Clusters of Orthologous Genes)	1.0	http://www.ncbi.nlm.nih.gov/ COG/	[4]
RefSeq	Taxonomically annotated bacterial and archaeal genomes	6/21/10	www.ncbi.nlm.nih.gov/refseq/	[5]
eggNOG	Functionally annotated orthologous proteins	2.0	http://eggnog.embl.de/	[6,7]
KEGG genes	Functionally annotated proteins	55.0/ 09-14	www.genome.jp/kegg/	[8,9]
NCBI NR	Non-redundant Proteins		ftp://ftp.ncbi.nlm.nih.gov/blast/db/	

Pipeline input

Data	Suffix	Description
Multiple fasta	.fasta	Metagenomic WGS sequences (1 file per sample)
Metadata	.txt	Sample-associated features (see section A for details)

Pipeline output

Data	Suffix	Description
UCLUST clusters	.clstr	List of clusters created to reduce redundant analysis
Replicate sequences	.txt	List of artificial 454 replicates removed from downstream analysis
BLAST hits	.raw	BLASTN or BLASTX to reference datasets results table ("-m 8" format)
Taxonomic assignments	.tsv	Table (tab-delimited) displaying taxonomic assignment counts for each sample
Functional assignments	.tsv	Table (tab-delimited) displaying functional assignment counts for each sample
Metastats output	.csv	Differentially abundant taxonomic or functional assignment groups (as pre-defined in Metadata input)
Skiff clustering	.pdf	Heatmap and two-way clustering based on taxonomic and functional assignment abundances
Pie charts	.pdf	Pie charts describing assignment abundances for up to 12 samples (not performed if >12 samples are given)
Stacked histograms	.pdf	Stacked histograms displaying relative abundances (up to 50 samples and 25 features)

A. Requirements for Pipeline Input

To run the full CloVR-Metagenomics analysis track, two different inputs have to be provided by the user: a set of fasta-formatted sequence files and a tab-delimited metadata file in the .txt format. The metadata file provides sample-associated information with the following formatting requirements:

#File	SampleName	ph	Description
A.fasta	sampleA	high	control
B.fasta	sampleB	high	sick
C.fasta	sampleC	low	treated
D.fasta	sampleD	low	treated

where:

1. All entries are tab-delimited.
2. All entries in every column are defined (no empty fields).
3. The header line begins with: #File<tab>.
4. There are no duplicate header fields or file names.
5. No header fields or corresponding entries contain invalid characters (only alphanumeric and underscore characters allowed)

Pairwise comparisons: To utilize the Metastats statistical methodology for the detection of taxonomic and functional assignments with differential abundance, the associated header field must end with "`_p`", (e.g. "`Treatment_p`", or "`ph_p`"). Otherwise Metastats will skip pairwise analysis.

B. Sequence clustering and artificial replicate removal with UCLUST

To reduce redundant database searches downstream, the UCLUST component of CloVR-Metagenomics first clusters all DNA sequences using a stringent 99% identity threshold. Similar to the procedure in [10], any non-representative sequence in a cluster that shares a prefix of length 8 with the representative (and whose length is within 10 bp of the representative's length) is determined to be an artificial 454 pyrosequencing replicate [11] and is removed from further analysis. Taxonomic and functional annotations made to representative members are later propagated to all non-replicate sequences.

C. Taxonomic assignment of DNA sequences

All representative DNA sequences from clusters are searched against the RefSeq database of finished prokaryotic genomes (by default) using BLASTN with the following options: "-e 1.0e-5" (e-value threshold), "-b 1" (number of alignments to show) and "-m 8" (tabular output). Each sequence is assigned to the taxonomy of the best-BLAST-hit.

D. Functional assignment of DNA sequences

All representative DNA sequences from UCLUST (section B) are searched against the COG database of orthologous gene groups (by default) using BLASTX with the same options as in section C ("-e 1.0e-5 -b 1-m 8"). Alternatively, the user may opt to employ

the KEGG genes, eggNOG or NCBI NR databases for functional annotation. Each sequence is assigned to the function of the best BLAST hit of the respective database.

E. Additional beta diversity analysis using Metastats and the R statistical package

E.1. Detection of differentially abundant features

The program Metastats uses count data from annotated sequences to compare two populations in order to detect differentially abundant features [3]. BLASTN results are processed to detect different taxonomic groups at multiple levels (phylum, class, order, family, genus), while BLASTX results are parsed for differentially abundant functional groups. Metastats produces a tab-delimited table displaying the mean relative abundance of a feature, variance and standard error together with a p value and q value to describe significance of the detected variations (see project website: http://metastats.cbcb.umd.edu/). Note Metastats can run analyses of 1 sample vs. 1 sample, or *N* samples vs. *M* samples, where *N* and *M* are greater than 1. It cannot perform a comparison of 1 sample vs. 2 samples.

E.2. Unsupervised sample clustering

Custom R scripts are used to normalize taxonomic or functional counts and subsequently calculate Euclidean-based distance matrices for samples and features. Complete-linkage (furthest neighbor) clustering is employed to create dendrograms of samples and taxa in the .pdf format. The R packages *RColorBrewer* and *gplots* are utilized.

E.3. Pie chart visualization

Custom R scripts are used to form pie charts displaying proportions of sequences assigned to specific functional and taxonomic levels for up to 12 samples. Outputs are in .pdf format. For more than 12 samples this function is not performed, as the visual comparison for the user would be cumbersome.

E.4. Stacked histogram visualization

Custom R scripts are used to form stacked histograms displaying proportions of sequences assigned to specific functional and taxonomic levels for up to 50 samples and 25 features. Graphical outputs are in .pdf format. For more than 50 samples or 25 features this function is not performed, as the visual comparison for the user would be difficult.

F. References

1. Edgar RC Search and clustering orders of magnitude faster than BLAST. Bioinformatics.

2. Altschul SF, Madden TL, Schaffer AA, Zhang J, Zhang Z, et al. (1997) Gapped BLAST and PSI-BLAST: a new generation of protein database search programs. Nucleic Acids Res 25: 3389-3402.

3. White JR, Nagarajan N, Pop M (2009) Statistical methods for detecting differentially abundant features in clinical metagenomic samples. PLoS Comput Biol 5: e1000352.

4. Tatusov RL, Fedorova ND, Jackson JD, Jacobs AR, Kiryutin B, et al. (2003) The COG database: an updated version includes eukaryotes. BMC Bioinformatics 4: 41.

5. Pruitt KD, Tatusova T, Maglott DR (2007) NCBI reference sequences (RefSeq): a curated non-redundant sequence database of genomes, transcripts and proteins. Nucleic Acids Res 35: D61-65.

6. Muller J, Szklarczyk D, Julien P, Letunic I, Roth A, et al. eggNOG v2.0: extending the evolutionary genealogy of genes with enhanced non-supervised orthologous groups, species and functional annotations. Nucleic Acids Res 38: D190-195.

7. Jensen LJ, Julien P, Kuhn M, von Mering C, Muller J, et al. (2008) eggNOG: automated construction and annotation of orthologous groups of genes. Nucleic Acids Res 36: D250-254.

8. Aoki-Kinoshita KF, Kanehisa M (2007) Gene annotation and pathway mapping in KEGG. Methods Mol Biol 396: 71-91.

9. Kanehisa M (2002) The KEGG database. Novartis Found Symp 247: 91-101; discussion 101-103, 119-128, 244-152.

10. Gomez-Alvarez V, Teal TK, Schmidt TM (2009) Systematic artifacts in metagenomes from complex microbial communities. Isme J 3: 1314-1317.

11. Quince C, Lanzen A, Curtis TP, Davenport RJ, Hall N, et al. (2009) Accurate determination of microbial diversity from 454 pyrosequencing data. Nat Methods 6: 639-641.

ClovR-Microbe: Assembly, gene finding and functional annotation of raw sequence data from single microbial genome projects – standard operating procedure, version 1.0

Kevin Galens, James Robert White, Cesar Arze, Malcolm Matalka, Michelle Gwinn Giglio, the CloVR team, Samuel V. Angiuoli & W. Florian Fricke

The Institute for Genome Sciences, University of Maryland School of Medicine, Baltimore, MD 21201, USA

Abstract
The CloVR-Microbe pipeline performs the basic processing and analysis steps required for standard microbial single-genome sequencing projects: A) Whole-genome shotgun sequence assembly; B) Identification of protein and RNA-coding genes; and C) Functional gene annotation. B) and C) are based on the IGS Annotation Engine (http://ae.igs.umaryland.edu/), which is described elsewhere [1]. The assembly component of CloVR-Microbe can be executed independently from the gene identification and annotation components. Alternatively, pre-assembled sequence contigs can be used to perform gene identifications and annotations. The pipeline input may consist of unassembled raw sequence reads from the Sanger, Roche/454 GS FLX or Illumina GAII or HiSeq sequencing platforms or of combinations of Sanger and Roche/454 sequence data. The pipeline output consists of results and summary files generated during the different pipeline steps. Annotated sequence files are generated that are compatible with common genome browser tools and can be submitted to the GenBank repository at NCBI.

Software

Step	Program	Version	Weblink	Reference
Assembly	Celera Assembler (CA)	5.4	http://sourceforge.net/apps/mediawiki/wgs-assembler/index.php?title=Main_Page	[2]
	Velvet	0.7.55	http://www.ebi.ac.uk/~zerbino/velvet/	[3]
Gene Finding	Glimmer	3.02	http://www.cbcb.umd.edu/software/glimmer/	[4]
	tRNAscan-SE	1.23	http://www.bioinformatics.org/wiki/TRNAscan-SE	[5]
	RNAmmer	1.2	http://www.cbs.dtu.dk/services/RNAmmer/	[6]
Annotation	Emboss	5.0	http://emboss.sourceforge.net/	[7]
	BLAST	2.2.21	http://blast.ncbi.nlm.nih.gov/Blast.cgi	[8]
	HMMer	2.3.2	http://hmmer.janelia.org/	[9]

Reference data

Database	Data	Version	Weblink	Reference
TIGRFAM	Protein families	7.0	http://www.jcvi.org/cms/research/projects/tigrfams/	[10]
Pfam	Protein families	22.0	http://pfam.sanger.ac.uk/	[10]
COG	Clusters of orthologous genes	1.0	http://www.ncbi.nlm.nih.gov/COG/	[11]
UniRef100	Proteins	Oct. 2010	http://www.ebi.ac.uk/uniref/	[12]

Pipeline input

Data	Suffix	Description
Raw sequence reads	.sff	454 sequencer output
Raw sequence reads	.fastq	Illumina sequencer output
Sequence reads	.fasta	Multiple sequence file
Sequence read qualities	.qual	Quality values corresponding to multiple sequence file
Pre-assembled contigs	.fasta	Multiple sequence file

Pipeline output

Data	Suffix	Description
Assembled contigs	.fasta	Multiple sequence file
	.bnk	Celera sequence assembly, compatible with Hawkeye [12] assembly viewer
Predicted genes	.coords	Coordinates table
	.fasta	Genes sequences (nucleotides)
	.pfasta	Protein sequences (amino acids)
Annotated sequences	.txt	Gene coordinates and annotation table
	.gbk	GenBank sequence file, compatible with Artemis [13] genome browser
	.tbl4sequin	Feature table for NCBI Sequin submission tool
	.xml	Machine-readable annotation file
Summary report	.txt	Pipeline summary report

A. Assembly

During the assembly process the fragmentary whole-genome shotgun (WGS) sequence data typically produced in microbial single-genome projects are used to reconstruct long contiguous sequences or *contigs*. Scaffolds are composed of multiple contigs that are linked by paired-end reads. In the assembly output each scaffold will be represented by a single sequence (FASTA) in which stretches of "N"s indicate gaps and estimated gap lengths spanned by paired-end reads.

A.1. Procedure

A.1.1. Assembly with the Celera Assembler

A.1.1.1 Raw sequence data file conversion

The Celera Assembler [2] uses .frg files as input, which are generated from 454 or Sanger raw sequences or from combinations of both data types with the sffToCA tool. Raw sequences from the 454 pyrosequencing platform are accepted either as single read or mated paired-end reads in the .sff format. Raw sequences from the Sanger platform are accepted as pairs of .fasta and .qual files with the same name prefix. As a default, sffToCA is run with the following parameters, which were optimized for data generated with the 454 GS FLX XLR Titanium platform: "-clear 454" sets the clear range for each sequence read "as is", using the clear range determined by the 454 sequencing machine; "-trim chop" erases sequences outside of the 454 clear ranges. If paired-end data are being used as input, a 454 linker has to be specified as either "-linker flx" or "-linker titanium", depending on the 454 sequencing platform generation that is being used. In addition, an insert size range has to be selected, e.g. "8000 1000" where the first number specifies the average insert length (8 kbp) and the second number the standard deviation (1 kbp). More than one .sff file can be used as input for sffToCA, which produces a single .frg file that serves as input for the assembly step.

A.1.1.2. Assembly

For the assembly process, Celera Assembler is run with the "runCA" executive script, using default parameters. Alternatively, a "spec file" can be provided by the user to select alternative parameters for the assembly process. Spec file examples can be downloaded from the Celera Assembler documentation page (http://sourceforge.net/apps/mediawiki/wgs-assembler/index.php?title=RunCA_Examples). The assembly step generates a number of different output files. First, assembly statistics are collected in a .txt summary report file. Second, .fsa (FASTA) files are generated as the direct assembly output, which serve as the input for the Gene Finding step. Third, a .asm file is generated, which can be used in combination with the .frg file from the sffToCA output to generate the .bnk file for visualization of the assembly results with the Hawkeye assembly viewer [13].

A.1.1.3. Celera assembly visualization

Using information from the raw sequence data (.frg) and the sequence assembly (.asm), a .bnk file is generated with the "toAmos" and "bank-transact" tools (http://sourceforge.net/apps/mediawiki/amos/index.php?title=Hawkeye). The .bnk file serves as the direct input for the Hawkeye assembly viewer [13], which itself is not part of the CloVR-Microbe pipeline and which is not installed on the CloVR virtual machine image.

A.1.2. Assembly with the Velvet Assembler

In the case of assembling Illumina-based WGS sequence data, the Velvet assembly program [3] can accept as input any combination of short or long read data (with or

without paired ends) in FASTQ or FASTA format. Additionally, start and end hash length parameters are required for kmer counting that Velvet performs, which must be odd values, and by default are "19" (start) and "31" (end). For paired-end data an insert-size and standard deviation must be provided, e.g. “-ins_length 300 -ins_length_sd 50”. Velvet outputs a .fasta file of all sequence contigs of at least 500 bp in length.

A.1.3. Split assembly into separate contigs
The following two steps of the CloVR-Microbe pipeline, B) Gene Finding and C) Annotation, are performed on each individual scaffold and/or contig from the WGS assembly separately. This is advantageous, as it allows for parallelization of processes.

B. Gene Finding

B.1. Identification of RNA genes

B.1.1. Identification of ribosomal RNA genes
Ribosomal RNA (rRNA) genes are identified with the RNAmmer tool [6]. Contig FASTA files (.fsa) are used as input to run RNAmmer with the "bac" option, which specifies "Bacteria" as the superkingdom of the input sequence source and the "lsu,tsu,ssu" option, which specifies the molecule types to search for as 5S/8S rRNA, 16S/18S rRNA, and 23S/28S rRNA. This step generates as output a .txt summary report file and gene coordinate files in the BSML, .xml, and .gff file formats, though these particular files are not downloaded at the end of the pipeline.

B.1.2. Identification of transfer RNA genes
Transfer RNA (tRNA) genes are identified with the tRNAscan-SE tool [5], which is run on FASTA files (.fsa) with the "-B" option to use the bacterial model for the prediction of tRNA genes. This step generates .txt coordinate file of all tRNA genes and a BSML (.bsml) file as output.

B.2. Identification of coding genes (CDS)

Open reading frames (ORFs) coding for proteins (CDS) are identified using the Glimmer3 software package [4]. The execution procedure is derived from the iterative self-training mode of operation described in the software documentation, which is available from the project website (http://www.cbcb.umd.edu/software/glimmer).

B.2.1. Identification of training set to build gene prediction model
The "long-orfs" program is run to identify long, non-overlapping ORFs from the total genome assembly, including all sequence scaffolds and/or contigs. These long ORFs serve as a training set to build the interpolated context model (ICM), which is used to predict CDS. The long-orfs program is run with the "-n" or "--no_header" option and the "-t 1.15" option, which specifies the cutoff for the entropy distance score used to select the training set of ORFs. The output is a list of non-overlapping ORFs.

B.2.2 Generation of interpolated context model
An interpolated context model (ICM) is built with data from the long-orfs output using the "build-icm" program with default options.

B.2.3 Glimmer3 gene prediction

B.2.3.1. First iteration

The first iteration of gene finding is run with the ICM model from B.2.2 using the "glimmer3" program executed with the following parameters: "-o50 -g110 -t30 -z11 -l -X". "-o50" sets the maximum overlap between CDS to 50 nucleotides; "-g110" sets the minimum CDS length to 110 nucleotides; "-t30" sets the threshold score for CDS to 30; "-z11" determines the NCBI translation table code "11" to specify stop codons; "-l" sets the input sequence as linear, and "-X" allows CDS to extend off the end of the input FASTA sequence.

B.2.3.2 Generation of Position Weight Matrix

A Position Weight Matrix (PWM) is generated for regions upstream of start-sites using the output from B.2.3.1. First, the script "upstream-coords.awk" is run with parameters '25 0' to extract sequence regions upstream of CDS predicted in B.2.3.1. Next, the "ELPH" program is run with parameter 'LEN=6' to create a PWM from the region upstream of the CDS start sites. ELPH is a general-purpose Gibbs sampler for finding motifs in a set of DNA or protein sequences (http://www.cbcb.umd.edu/software/ELPH/). The distribution of start codons is also generated from the output of the first iteration.

B.2.3.3 Second iteration

The second iteration of glimmer3 gene finding is run with the ICM from B.2.3.1 and the PVM from B.2.3.2 with parameters "-o50 -g110 -t30 -z11 -l –X –P <number-list>". <number-list> consists of three comma-delimited values that specify the probabilities of different start codons as determined from the output of B.2.3.2. The output includes a set of putative CDS described in a .txt summary report and coordinate files of all predicted genes in the BSML format.

C. Annotation

C.1. Translation of preliminary CDS into peptides

All predicted protein-coding genes from all scaffolds and/or contigs are translated into peptide sequences using a wrapper to the "transeq" program from the EMBOSS package [7] with the parameters "-table 11" to specify the bacterial translation table. The "-trim 1" option is also used to eliminate trailing "X" or "*" characters from the translation. A peptide FASTA file (.fsa) is provided as the output.

C.2. CDS homology searches (round 1)

Two types of homology searches are performed to generate the evidence, which is used to assign a functional annotation to each CDS: the translated CDS are compared against the UniRef100 non-redundant protein database from UniProt (http://www.uniprot.org/) using BLASTX, and against the two protein family databases Pfam and TIGRFAM [10], using HMMER [9].

C.2.1. Protein comparison and pairwise alignment

For this step, the Blast-Extend-Repraze (BER) tool (http://sourceforge.net/projects/ber/) employs a two-step process that starts with a BLASTX search followed by a modified Smith-Waterman alignment. The BER output is used to detect possible frameshifts and in-frame stop codons within the predicted CDS.

C.2.1.1. BLASTX protein comparison

The following parameters are used to perform BLASTX comparisons of all translated CDS against UniRef100: "-e 1e-5" (e-value cut-off), "-F T" (filter for low complexity regions), "-b 150" (show alignments for 150 database sequences), "-v 150" (show one-line descriptions for 150 database sequences), "-M BLOSUM62" (use BLAST matrix BLOSUM62).

C.2.1.2. Modified Smith-Waterman nucleotide sequence alignment

In order to identify frameshifts, in-frame stop codons and erroneous start codons the BER tool creates nucleotide sequence alignments of the CDS and the nucleotide sequence corresponding to the protein matches identified in C.2.1.1. For these sequence alignments the CDS are extended by 300 nucleotides upstream and downstream of the start and stop codon. Therefore, if there is a sequencing error or a natural mutation that has split one gene into two, the BER tool creates an alignment across those two fragments. BER is executed with the following parameters "-e 1e-5" (maximum e-value), -E 1e-5 (maximum p-value), "-n 150" (maximum number of hits, similar to the BLAST -v option)), "-N 0" (maximum number of hits per region).

C.2.2. Protein family comparisons

Each translated CDS is searched against two database of Hidden Markov Models (HMMs) of protein and protein domain families, TIGRFAM (http://www.jcvi.org/cms/research/projects/tigrfams/) and Pfam (http://pfam.sanger.ac.uk/), using HMMER2 [8] with default parameters.

C.3. CDS overlap analysis

The results from the tRNA, rRNA and CDS gene calls together with the evidence generated through the HMM homology searches are used to remove overlapping genes either between CDS or between CDS and tRNA or rRNA genes. Only overlaps of at least 60 nucleotides are considered for resolution. When a CDS that has no homology evidence (HMM matches passing cutoff or BER alignments) overlaps with another CDS that does contain evidence, the CDS without evidence is removed. If both (or neither) of the CDS' show homology evidence, they are left in place. When a CDS overlaps with an RNA prediction, the RNA prediction is given a higher priority and the CDS is removed.

C.4. CDS homology searches (round 2)

After the automatic curation of start sites, the newly changed gene models are retranslated. These new polypeptides are then run through another set of BLASTX, HMM and BER searches to update similarity evidence for functional annotation. In addition, each polypeptide is run against the NCBI COG database using BLASTP with parameters "-e 1e-5 -F 'F' -b 500 -v 500 -M BLOSUM62".

C.5. Functional annotation

An in-house script is used to assign functional names, gene symbols, EC numbers and GO terms to each CDS based on a ranked hierarchy of evidence sources generated through the homology searches. The script considers sequence homology to TIGRFAM and PFAM HMMs and matches to the UniRef100 protein database. BER evidence is used to identify searches against UniRef100 with at least 35% sequence identity over 80% gene length. A series of naming rules are applied based on the type of HMM match. Equivalog HMM hits are most preferred and names are used without modification. For other HMM types protein names are appended with 'family protein' or 'domain protein' based on the isology type of the HMM. If the protein matches a

hypothetical equivalog HMM the name will be ‘conserved hypothetical protein'. The decision process that is used to determine functional annotations is identical to the one from the IGS Annotation Engine, which is described in detail in a publication elsewhere [1].

C.5. Creation of different output files

To allow users of the pipeline to edit, visualize, and publish the annotated sequences and to submit them to the public sequence databases, the pipeline output is stored in a number of different file formats. For each annotated sequence assembly or contig .gbk and .gff files are generated, which can be opened and edited with the Artemis sequence annotation tool [14]. A .txt feature file can be loaded into the Sequin tool and used for GenBank sequence submission (http://www.ncbi.nlm.nih.gov/Sequin/index.html).

D. References

1. Galens K, Orvis J, Daugherty S, Creasy HH, Angiuoli S, et al. (2011) The IGS Standard Operating Procedure for Automated Prokaryotic Annotation. Stand Genomic Sci. 4(2):244-51.

2. Miller JR, Koren S, Sutton G (2010) Assembly algorithms for next-generation sequencing data. Genomics 95: 315-327.

3. Zerbino DR, Birney E (2008) Velvet: algorithms for de novo short read assembly using de Bruijn graphs. Genome Res 18: 821-829.

4. Delcher AL, Bratke KA, Powers EC, Salzberg SL (2007) Identifying bacterial genes and endosymbiont DNA with Glimmer. Bioinformatics 23: 673-679.

5. Lowe TM, Eddy SR (1997) tRNAscan-SE: a program for improved detection of transfer RNA genes in genomic sequence. Nucleic Acids Res 25: 955-964.

6. Lagesen K, Hallin P, Rodland EA, Staerfeldt HH, Rognes T, et al. (2007) RNAmmer: consistent and rapid annotation of ribosomal RNA genes. Nucleic Acids Res 35: 3100-3108.

7. Rice P, Longden I, Bleasby A (2000) EMBOSS: the European Molecular Biology Open Software Suite. Trends Genet 16: 276-277.

8. Altschul SF, Madden TL, Schaffer AA, Zhang J, Zhang Z, et al. (1997) Gapped BLAST and PSI-BLAST: a new generation of protein database search programs. Nucleic Acids Res 25: 3389-3402.

9. Eddy SR (2009) A new generation of homology search tools based on probabilistic inference. Genome Inform 23: 205-211.

10. Haft DH, Selengut JD, White O (2003) The TIGRFAMs database of protein families. Nucleic Acids Res 31: 371-373.

11. Tatusov RL, Fedorova ND, Jackson JD, Jacobs AR, Kiryutin B, et al. (2003) The COG database: an updated version includes eukaryotes. BMC Bioinformatics 4: 41.

12. Suzek BE, Huang H, McGarvey P, Mazumder R, Wu CH (2007) UniRef: comprehensive and non-redundant UniProt reference clusters. Bioinformatics 23: 1282-1288.

13. Schatz MC, Phillippy AM, Shneiderman B, Salzberg SL (2007) Hawkeye: an interactive visual analytics tool for genome assemblies. Genome Biol 8: R34.

14. Carver T, Berriman M, Tivey A, Patel C, Bohme U, et al. (2008) Artemis and ACT: viewing, annotating and comparing sequences stored in a relational database. Bioinformatics 24: 2672-2676.
